# Firearm-related suicides, homicides, and homicide-suicides involving security officers in two East African Countries: a press media review

**DOI:** 10.1186/s12888-023-05368-6

**Published:** 2023-11-24

**Authors:** Moses Muwanguzi, Moses Kule, Simpson Nuwamanya, Mark Mohan Kaggwa

**Affiliations:** 1 Department of Psychiatry, Faculty of Medicine, Mbarara University of Science and Technology, Mbarara, Uganda; 2 Department of Psychiatry, Mbarara Regional Referral Hospital, Mbarara, Uganda; 3 Department of Psychiatry and Behavioural Neurosciences, McMaster University, Hamilton, Canada; 4 Forensic Psychiatry Program, St Joseph’s Healthcare Hamilton, 100 West 5Th, Hamilton, ON L89 3K7 Canada

**Keywords:** Suicide, Homicide, Uganda, Kenya, East Africa, Security officers, Gun violence, Firearms, Media report review

## Abstract

**Background:**

Firearm violence is a growing public health problem causing death globally. With easy accessibility to firearms, suicides, homicides, and homicide-suicides have increased among security officers, especially in developing countries affected by long-standing civil wars/political insurgencies. No study has explored firearm violence in East African countries. This study describes the press media reporting of suicides, homicides, and homicide-suicides among security officers in two East African countries (Uganda and Kenya).

**Methods:**

Due to the absence of suicide databases among East African countries, the present study reviewed press media reports. We utilized content analysis of suicides, homicides, and homicide-suicides reports among security forces. Relevant media reports between January-2020 and May-2023 were searched. Using ANOVA and chi-square tests, we tested for statistical differences in characteristics between victims and perpetrators.

**Results:**

Among the 56 perpetrated reports, most of them were homicides 44.64% (n = 25/56), 30.36% (n = 17/56) were homicide-suicides, and 25% (n = 14/56) were suicides. Perpetrators’ age ranged from 21 to 47 years, majority being males [53/56 (94.64%)]. Victims were 58, mostly Ugandans [41/58 (73.21%)] with a mean age of 33.5 ± 8.81 years. Among the three main outcomes, statistically significant difference existed by country (χ^*2*^ = 23.88, *p* < 0.001), and perpetrators’ age (F = 8.59, *p* = 0.005). There was a significant difference between perpetrators and the number of victims lost by age of victims (F = 10.37, *p* = 0.002). Among victims, type of security of perpetrator and citizenship of victims (χ^2^ = 24.18, *p* < 0.001) showed statistical difference with Ugandans having more victims to army officers while Kenyans to police officers. Brief incident descriptions pointed towards relationship dysfunctions, alcohol/substance abuse, intentional harm, and financial disagreements, as the potential causes. Only two perpetrators were reported to have mental health-related conditions.

**Conclusion:**

This study shows that media reported firearms-related suicides, homicides, and homicide-suicides among security forces commonly involve males. Perpetrators in Uganda are mainly army officers while in Kenya the perpetrators are mostly police officers. Mental health conditions were not frequently reported among perpetrators. We recommend strengthening and enforcing gun regulation policies among security officers to curb this growing problem in these countries. Routine screening of mental health problems to enable early interventions is recommended among security officers.

**Supplementary Information:**

The online version contains supplementary material available at 10.1186/s12888-023-05368-6.

## Introduction

Firearm violence is a widely spreading and devastating preventable public health problem [1, 2]. In 2016, over 250,000 preventable deaths worldwide were due to firearm-related violence [3]. However, in the past two decades prior to COVID-19, unintentional use of firearms has claimed more than 2·75 million lives [4]. A sharp rise in firearm-related intentional and unlawful deaths (suicide and homicide) were noted in many parts of the world following the COVID-19 pandemic [5]. Firearm-related deaths contribute a substantial burden on health care and economic systems, with permanent public psychological trauma [4]. Approximately, 225 years of life per 100,000 people are lost to disability and premature death as a result of gun violence [6].

The use of firearms as a mechanism of suicide or homicide is largely based on the availability of firearms [7, 8]. No wonder, firearm-related suicide and/or homicide are frequent among security forces who have easier access to firearms [9]. In countries that have been recently involved in wars or insurgencies, firearm access increases, and these are followed by an increase in firearm-related violence, such as suicide and homicide [10]. Many developing countries, especially in Africa, have had prolonged periods of political insurgencies. However, many of these countries have no dedicated databases to track these deaths [1, 3].

East Africa is one of the regions on the African continent that has seen multiple civil wars. However, none of the countries has a database to track firearm-related deaths. We therefore used press media reports to show the burden of firearm-related death involving security officers. Generally, there is scarcity of literature about suicides/homicides among security officers even though there are recently more press media reports about suicides/homicides involving security officers [11–16]. These media reports insinuate different public reactions and the need for firearms policy changes. Therefore, we based on the available press media information to analyze the likely public perceptions and try to identify the various relationships between the victims and the perpetrators (security officers). Our working hypothesis was that a country in East Africa that has been affected with more insurgencies/wars may have more firearm-related deaths. We therefore compared two countries, i.e., Uganda and Kenya.

Press media reports have been used widely as a source of gray data on different topics, especially suicide in the general population like in Uganda, which has yielded tremendous informative discussions [17, 18]. The use of press media as a source of gray data is especially important in understanding topics that might have triggered public attention.

### Uganda and Kenya security officers

These two East African countries have both private and public security agencies that are legislated and regulated by the national constitution of the respective republics. Private security organizations (PSOs) have non-commissioned security personnels mandated to protect lives and properties of citizens or organizations that can afford their services [19]. The public/governmental security bodies (police, army, and prisons) are intrinsically organized by experience into commissioned and noncommissioned service categories [20]. This contributes to the leadership structure that supports the functionality of these security forces in a more organized way. The ranking system of the security personnels in the two countries are similar because the two countries were former British colonies.

The security forces are organized as follows: (I) the army (Uganda People’s Defense Forces [UPDF] and Kenya Defense Forces [KDF]) whose role is to preserve and defend the sovereignty and territorial integrity of the countries [21, 22]; (ii) police (Uganda Police Force [UPF] and National Police Service [NPS] of Kenya) whose role is to protect life, property, law and order [23], in addition to maintaining professionalism, prevent corruption, maintain freedom, and dignity [24]; and (iii) others, including the Uganda Prisons Service (UPS), and Intelligence Services (IS). All security agencies are legislated by the parliament, having the president as the commander-in-chief [25]. Each security agency is mandated to serve within its jurisdiction as provided for by the law/constitution.

Depending on the entry training level into police, the lowest qualification to join security forces in Uganda is a primary level certificate (for local defense unit) [20]. On the other hand, recruitment into Kenyan defense forces is a rigorous process with a minimum acceptable academic qualification for the first level of general service officer being a grade B, with C + in English, Mathematics and one science subject [26]. The training period for this category covers three years leading to award of the bachelor of science in Military Science and Security Studies upon completion [26], which is not the case in Uganda.

### Security threats of the two states

Uganda and Kenya have existed in harmony with no reported history of inter-state conflicts. However, both states have actively participated in political conflicts of neighboring states majorly South Sudan, and counter-fighting rebel groups in Somalia (especially the Al-Shabaab) and democratic republic of Congo (especially the Allied Democratic Forces-ADF) till to-date [27–29]. The aftermaths of such attacks are a significant source of psychological trauma to the security forces and public [29].

### Gun regulation for security personnel in the two countries

The deployment of firearms at large scale to counterattack the insurgencies in the region led to establishment of firearms and gun regulations to control possession of firearms. The constitution for both countries provides for a strict and regulated use of firearms among security forces through the ‘**Firearm act**’ specific to each country [30]. In addition, there are additional laws of firearm use legislated under laws that manage each government’s security agencies, for example the Police act Part V Sect. 28 stipulates the *‘use of firearms by police officers in special cases*’ [31]. The Firearms act in Uganda (1970) is more recent compared to that of Kenya (1954) and the relevant key differences are briefly described in the Table 1.﻿

**Table 1:** Key firearm regulatory constitutional areas to be considered in suicides, homicides, and suicide-homicides among security officers in Uganda and Kenya

Key regulatory areas	Uganda’s constitution as promulgated on October 1995	Kenya’s constitution as promulgated on August 2010
Regulatory act	Firearms act (1970)	Firearms act (1954)
Carrying firearm while drunk or disorderly	Any person who, whether by reason of drinking or otherwise, while carrying a firearm, acts in a dangerous or disorderly manner and commits an offence, is liable to imprisonment for a period not exceeding six months or to a fine not exceeding two thousand shillings (approximately half a US dollar) or to both	Any person who is drunk, or who behaves in a disorderly manner, while carrying a firearm, shall be guilty of an offence and liable to imprisonment for a term not exceeding one year or to a fine not exceeding ten thousand Kenyan shillings (approximately 70 US dollars) or to both
Penalty for unlawful use of firearms by security officers	Where a person subject to the code commits or attempts to commit an offence against the code, he or she may be arrested with or without a warrant by a police officer higher in the rank	For unlawful use of firearm by police officers, shall be guilty of an offence, and liable to imprisonment for a term of not less than seven years and not more than fifteen years and shall, in addition, be automatically dismissed from the public service and, subject to Sect. 113 of the Constitution, forfeit all rights to any pension, gratuity or other payment which may at the date of his conviction have accrued due to him

Currence convergence based on Google Finance on October 8, 2023

Background checks including criminal records, mental illness, addictions, and domestic violence are required before someone gets a firearm [32, 33]. In Kenya, domestic violence perpetrators can have their firearm license evoked [33].

### The current study

This study has employed a more public-sensitive approach through press media records review to describe suicides, homicides, and homicide-suicides among security forces in the two East African countries (Uganda and Kenya). We have also provided a brief qualitative assessment of the incident descriptions to better understand the triggers and potential causes that would have led to the shootings.

## Methods

### Study area and design

This press media review captured homicides/suicides/homicide-suicides published by online press media websites from January-2020 to May-2023 in Uganda and Kenya. This was a search of gray data that was publicly available, thus no ethical approval was required. We assessed online press media reports, with the notion that “*what the media publishes, is what the public perceives*”. Most of these reports were media accounts of the spokesperson of the respective security agencies. In addition, on-scene experiences were provided by area residents, local security personnels, family, friends, neighbors, and/or others who knew the victim(s) before the incident. All this information was conjunctively considered when reporting each incident and victim(s).

### Search strategy

Using this method, data were searched and collected from the different press media websites published in Uganda and Kenya between January 2020 to May 2023 (40 months). This corresponds with the pre, during, and the post COVID-19 lock down periods—periods in which many East African countries were restructuring their entire health and economic systems impacted by COVID-19 and lockdown related adverse effects [34].

Searches were done both on *“Google”* and within the press media websites. The following key words were used “gun”, “shot”, “suicide”, “murder-suicide”, “homicide”, “police” and “security”, “Kenya”, “Uganda”.

### Selection criteria

After running the search terms, all press media articles that reported homicides, suicide, homicide-suicides/murder-suicides where a security officer was involved in the illegal use of a firearm to shoot self (suicide), others (homicide), or both (homicide-suicide) were considered. All recorded incidents resulted into either death of the perpetuator (suicides), the victim(s) (homicides), or both perpetrator(s) and victim(s) (homicide-suicides).

### Data abstraction and quality assurance

After thorough review of the articles, information collected was categorized into two sections A and B. Section A described the security officers (perpetuators) who used the firearm(s) to cause loss of lives, whereas section B recorded information about the victim(s) who succumbed during the incidents through homicides or homicide-suicides.

Information captured in section A included; *a)* country, *b)* year, *c)* month, *d)* time of incident [day/night], *e)* URL of the article, *f)* form of incident [homicide/suicide/homicide-suicide], *g)* sex of perpetuator [male/female], *h)* age of perpetuator [in years], *a)* marital status of perpetuators [married/unmarried], *j)* type of security of the perpetuator [police officer/army officer/security guards/others], *k)* service category of the perpetuator [non-commissioned/junior/senior], and *m)* perpetrator’s employer [government/private].

Section B consisted of victim(s)’s characteristics, including *a)* nationality of the victim [Ugandan/Kenyan/others], *b)* age of victim [in years], *c)* sex of the victim [male/female], *d)* victim(s)’ official category [civil/security officer], *e)* victim’s occupation, *f)* relationship of victim with the perpetuator, and *g)* place of death.

The final extracted tables have been presented; see Supplementary Table 1 (for security officers – perpetrators) and Supplementary Table 2 (for victims). The detailed ranks of the service categories of the perpetuators have been categorized in Supplementary Table 3.

Data were collected by three members of the research team (MM, MMK, and MK) competent in most official languages used by Kenya and Uganda.

### Operational definitions

#### Victim

Victim was defined as an individual (whether a security officer or civilian) who was killed in homicides or homicide-suicides incidents. Therefore, security officers who died by suicide using guns were not considered victims.

#### Perpetuator

Perpetuator was defined as a security officer who used a firearm to cause loss of a life, either by killing other(s) (in homicides), to kill self (in suicides), or both (in homicide-suicides).

### Data management and analysis plan

**Fig. 1:**
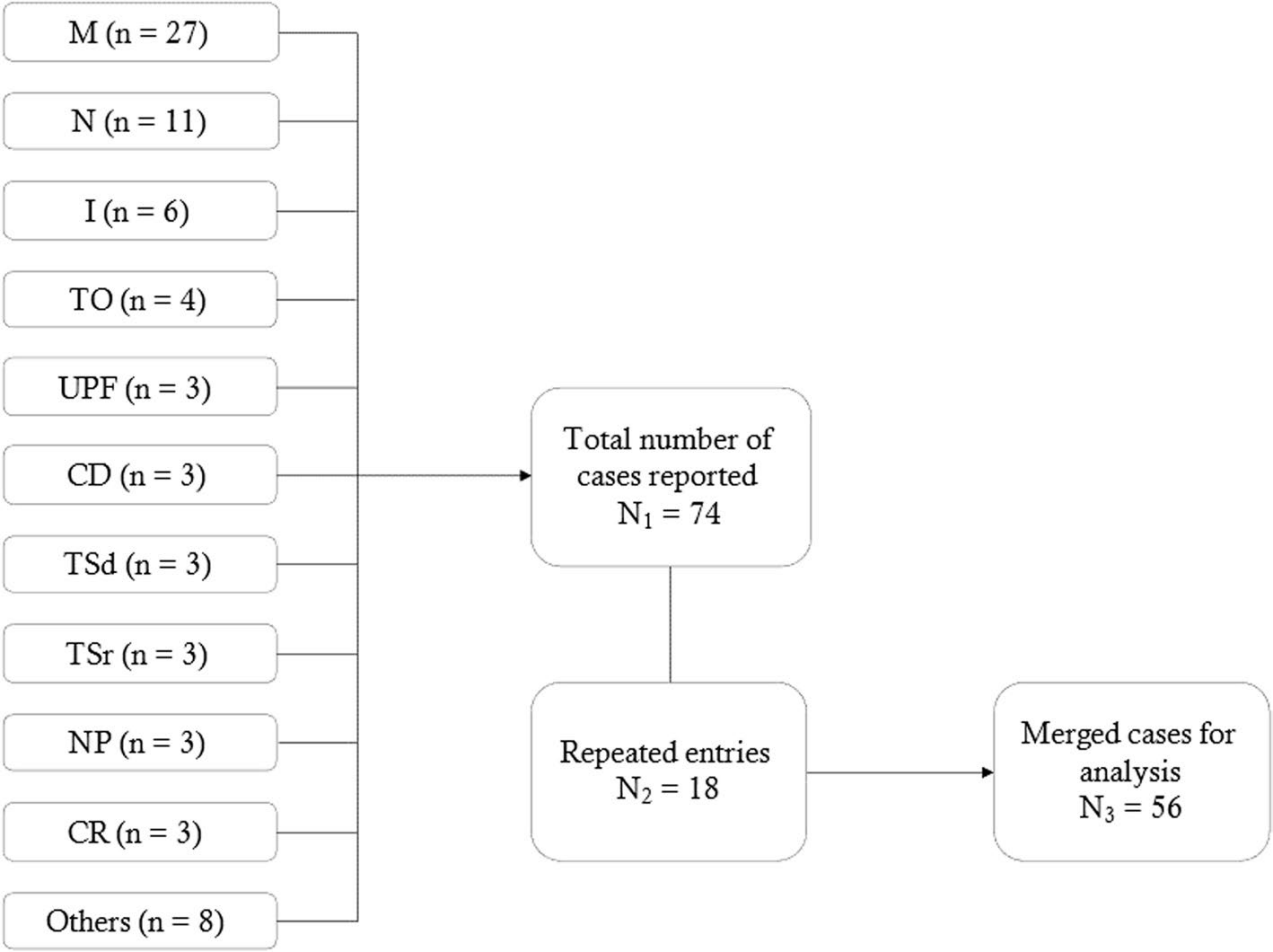
The flow diagram showing press media reports from different media websites. Key: M = Monitor, N = Nation, I = Independent, TO = The Observer, UPF = Uganda Police Force, CD = Citizen Digital, TSd = The Standard, TSr = The Star, NP = Nile Post, CR = Chimb Reports, Others = These were 8 sites which had only recorded one incident per media site; *Tuko, Radio-Rukungiri, Red Pepper, Aljazeera, NTV Uganda, Flash Media Uganda,* and *Daily Express*

Data was collected using *Google forms* after which an automatic spreadsheet was generated. Data cleaning was done in *Microsoft Excel* spreadsheet 2019. Similar records from different websites were merged to supplement information to the incidents (Fig. 1).

Data was analyzed using STATA V.15 [35]. Descriptive statistics were presented using frequencies and percentages (for categorical variables) and mean and its standard deviations (for normally distributed continuous variables like age of victims). Analysis of variance (ANOVA) and Pearson’s Chi-square statistics were performed to identify differences amongst perpetuators (Table 2), and victims (Table 3). Additionally, Table 4 showed the differences in the cadres of perpetrators in relation to the victims.

**Table 2:** Differences in characteristics among perpetrators involved in the different forms of suicide/homicides (*N* = 56)

Perpetrators characteristics	Total N (%)	Forms of incidents			F/χ^2^ (*p-*value)
		**Homicides** **n (%)** **25 (44.64)**	**Suicides** **n (%)** **14 (25.00)**	**Homicide-suicides** **n (%)** **17 (30.36)**	
**Country**					
Kenya	15 (26.79)	2 (13.33)	1 (6.67)	12 (80.00)	23.88 (< 0.001)
Uganda	41 (73.21)	23 (56.10)	13 (31.71)	5 (12.20)	
**Age (μ ± SD); missing**	33.5 ± 8.81; 41	28.80 ± 6.14; 20	44.25 ± 2.99; 10	30.17 ± 7.33; 11	8.59 (0.005)
**Sex**					
Female	3 (5.36)	1 (33.33)	1 (33.33)	1 (33.33)	0.19 (0.910)
Male	53 (94.64)	24 (45.28)	13 (24.53)	16 (30.19)	
**Type of security**					
Police officers	29 (51.79)	10 (34.48)	8 (27.59)	11 (37.93)	8.00 (0.238)
Army officers	18 (32.14)	9 (50.00)	5 (27.78)	4 (22.22)	
Security guards	6 (10.71)	5 (83.33)	1 (16.67)	0 (0)	
Others	3 (5.36)	1 (33.33)	0 (0)	2 (66.67)	
**Service categories of officers**					
Non-commissioned	30 (52.63)	14 (46.67)	7 (23.33)	9 (30.00)	2.17 (0.705)
Junior	4 (7.02)	1 (25.00)	2 (50.00)	1 (25.00)	
Senior	4 (7.02)	1 (25.00)	1 (25.00)	2 (50.00)	
Missing	19 (33.33)	-	-	-	
**Employment**					
Government	47 (83.93)	19 (40.43)	12 (25.53)	16 (34.04)	2.51 (0.286 0.286)
Private	9 (16.07)	6 (66.67)	2 (22.22)	1 (11.11)

**Table 3:** The relationship between victims’ characteristics and types of security of perpetrators

Victims’ characteristics	Type of security					
	**N (%)**	**Police officer** **n (%)** **29 (50.88)**	**Army officer** **n (%)** **21 (36.84)**	**Security guards** **n (%)** **5 (8.77)**	**Others** **n (%)** **2 (3.51)**	**F/χ **^**2**^** (*p*-value)**
**Citizenship of victims**						
Ugandans	34 (59.65)	9 (26.47)	19 (55.88)	4 (11.76)	2 (5.88)	24.02 (**0.001**)
Kenyans	20 (35.09)	18 (90.00)	2 (10.00)	0 (0)	0 (0)	
Others	3 (5.26)	2 (66.67)	0 (0)	1 (33.33)	0 (0)	
**Age of victims**						
Mean ± SD	32.72 ± 10.94	30.70 ± 8.81	35.45 ± 16.11	28.67 ± 4.04	24 ± 0	0.32 (0.967)
**Gender**						
Females	20 (35.09)	9 (50.00)	8 (40.00)	2 (10.00)	1 (5.00)	0.54 (0.910)
Males	37 (64.91)	20 (54.05)	13 (35.14)	3 (8.11)	1 (2.70)	
**Victim category**						
Civilian	18 (31.58)	12 (66.67)	5 (27.78)	0 (0)	1 (5.56)	5.04 (0.169)
Officer	20 (35.09)	8 (40.00)	7 (35.00)	4 (20.00)	1 (5.00)	
Missing	19 (33.33)	-	-	-	-

**Table 4:** The relationship between the perpetrators’ characteristics and the number of victims lost

Perpetrators’ characteristics	Number of victims lost			
	**None** **n (%)** **15 (26.79)**	**One victim** **n (%)** **34 (60.71)**	**More than one victim** **n (%)** **7 (12.50)**	**F/χ**^**2**^** (*p-*value)**
**Country**				
Kenya	1 (6.67)	11 (73.33)	3 (20.00)	4.56 (0.103)
Uganda	14 (34.15)	23 (56.10)	4 (9.76)	
**Age (μ ± SD); missing**	44.25 ± 2.99; 11	29.10 ± 6.69; 24	34; 6	9.16 (**0.004**)
**Sex**				
Female	1 (33.33)	1 (33.33)	1 (33.33)	1.54 (0.462)
Males	14 (26.42)	33 (62.26)	6 (11.32)	
**Type of security**				
Police officers	9 (31.03)	16 (55.17)	4 (13.79)	4.68 (0.585)
Army officers	5 (27.78)	10 (55.56)	3 (16.67)	
Security guards	1 (16.67)	5 (83.33)	0 (0)	
Others	0 (0)	3 (100.00)	0 (0)	
**Service categories of officers**				
Non-commissioned	8 (26.67)	17 (56.67)	5 (16.67)	2.27 (0.685)
Junior	2 (50.00)	2 (50.00)	0 (0)	
Senior	1 (25.00)	3 (25.00)	0 (0)	
Missing				
**Employment**				
Government	13 (27.66)	27 (57.45)	7 (14.89)	2.36 (0.308)
Private	2 (22.22)	7 (77.78)	0 (0)	

## Results

The total press media incidents captured were 74, from which 17 were repeated entries of similar incidents from different press media websites [11–16, 36–104]. After merging such repeated entries, we identified a total of 56 incident reports (Fig. 1). It is important to note that the number of perpetrators was 56. Since some security officers killed more than one victim the total number of victims was higher than perpetrators (n = 58).

The majority of the incidents were homicides [n = 25/56 (44.64%)], followed by homicide-suicides [n = 17/56 (30.36%)], and only 14/56 (25.00%) were suicides. Of all these incidents, majority were from Uganda [41/56 (73.21%)] with only 15/56 (26.79%) from Kenya. There was a steady rise in the number of incidents per year from January-2020, where the least [9/56 (16.07%)] occurred in 2020, 13/56 (23.21%) in 2021, and most of the incidents were in 2022 [23/56 (41.07%)], and only 11/56 (19.64%) in 2023 by May, see Table 2.

### Characteristics for security officers and their differences among those involved in the different forms of incidents (*N* = 56)

Ages of perpetuators ranged from 21 to 47 years with the mean age of 33.5 ± 8.81 years, most were males [53/56 (94.64%)], majority of whom were police officers [29 (51.79%)], mostly working as non-commissioned officers [30/38 (78.95%)], and over four-fifth working for the government [47 (83.93%)]. Furthermore, only 2 officers were reported to have history of mental illness.

The relationship between the country and the form of incidents was statistically significant (χ^*2*^ = 23.88, *p* < 0.001), in which perpetrators involved in homicides and suicides were predominantly from Uganda, whereas those involved in homicide-suicides were mostly from Kenya. In addition, perpetrators who died by suicide were statistically significantly older with the mean age of 44.25 ± 2.99 years compared to others, that is 30.17 ± 7.33 years for those involved in homicide-suicides and those involved in homicides being the youngest, 28.33 ± 5.61 years. This relationship was also statistically significant (*F* = 8.59, *p* = 0.005), see Table 2.

### Characteristics of victims and their differences among those who were killed by different types of security officer (*N* = 58)

The total number of victims was 58. Victims who succumbed to these incidents were mostly Ugandans [35/58 (60.34%), with a mean age of 32.28 ± 12.19 years. About two-thirds [38/58 (65.52%)] were males, majority being security officers [20/58 (35.09%)]. Most of the incidents with reported marital status were married [25/26 (96.15%)] with only one [1/26 (3.85%)] who was single/unmarried. The relationship between citizenship of victims and the cadres of perpetrators involved in the incidents was statistically significant (χ^*2*^ = 24.18, *p* < 0.001). It was noted that army officers were involved in more incidents among mostly Ugandan victims, whereas police officers and to a little extent security officers were implicated among Kenyans, see Table 3.

### The relationship between the perpetrator’s characteristics and the number of victims lost in the incidents

Security officers who had no victims lost (involved in suicide alone) were older [44.25 ± 2.99 years] than those with victims (i.e., 28.82 ± 6.42) years and 34 years for those with one victim and more than one victim, respectively (*F* = 10.37, *p* = 0.002), see Table 4.

### Common themes about the incidences

From the brief incident descriptions in Supplementary Table 1, major themes were derived to describe the possible triggers to the shootings, and these have been described below.

#### Relationship dysfunctions

Most of the incidents existed between security officers and victims who existed in toxic and dysfunctional intimate relationships as lovers, girlfriends/boyfriends, and husband/wives. In some incidents, there were reports of allegations of infidelity and marital disagreements among the two parties which frequently sparked off heated arguments that later resulted into shooting [11–13, 63, 67, 68, 70, 72, 73, 82, 84–91, 96, 101]—incidents 12, 23, 27, 28, 30, 32, 38, 40, 41, 42, 43, 44, 49, and 55. Some reported inter and intra-family relationship disputes that might have precipitated into shooting, like incidents 52 [99, 100].

#### Alcohol and substance use

Many of the incidents were described to have happened at entertainment places moreover at night, especially bars and restaurants where perpetrators were reportedly drinking alcohol. In many incidents, it is frequently reported that some perpetrators acted under obvious alcohol intoxication. Some police officers were reported to have been drinking alcohol all-day long, prior to the incidents and others had visited bars illegally during COVID-19 lockdown to booze [54, 61, 62, 94, 101]—incidents 15, 22, 47, and 53.

#### Intentional harm

To a large extent, most incidents were perpetrated voluntarily with pre-planned intent. These perpetrators were reported to have had a prior plan or made an informed decision that guided their act of shooting. For instance, in one incident (case 13) [51, 52], after a heated argument, the perpetrator promised to return with vengeance where he later returned with a firearm, shot and killed the victim in a homicide. In other Incidents [49, 53, 58, 59, 82, 86–88, 95, 97, 101, 104], the perpetrators had to halt normal daily activities or change course of activities to perform the act—incidents 10, 14, 19, 20, 38, 42, 48, 50, 53, and 56. In addition, individuals who left a suicide note(s) showed their suicidal act had been pre-planned in advance as in incidents 17 [56] and 37 [81]. In many cases, the perpetrators illegally carried the guns away from their duty stations with intent to cause harm as for incidents 1 [37] and 34 [76].

#### Financial disagreements

Some incidents happened due to inability to reach agreement about financial payments while some perpetrators reported financial challenges with allegations of delayed salary payments by the victim, as in incident 5 [41, 42]. For example, in incident 1 the perpetrator had disagreements over loan payment terms with the civilian victim, that resulted in shooting and instant death of the victim. Similarly, for incident 16, there was reported disagreements about the transport fares, that triggered off shooting.

#### The superiority-inferiority complex

In several incidents, perpetrators who were instigated for misbehavior (like late coming, becoming drunk at work) by their seniors, took offence and decided to shoot their seniors in retaliation, as described for incidents 29 [69] and 36 [80].

## Discussion

The present study investigated the press media report evidence of firearm related suicides, homicides, and homicide-suicides involving security officers in two East African countries i.e., Uganda and Kenya. We describe the characteristics of the perpetrators and the victims involved in suicides, homicides, and homicide-suicides as described by press media reports. The reasons for the findings are discussed under the subheadings below.

### Suicides, homicides, and homicide-suicides among Ugandan and Kenyan security forces

In the present study, about three-quarters (73.21%) of the incidents were from Uganda as compared to Kenya. This significant difference may be due to differences in the political environment and enforcement of firearm regulations among security officers between the two countries. Kenya has very stringent penalty for unlawful use of firearms among security officers including, imprisonment for a term of not less than seven years and not more than fifteen years, automatic dismissal from the public service, a higher pay in fines, forfeit to all rights to any pension, gratuity or other payments which may at the date of this conviction have accrued due to him, see details in Table 1 [105]. Therefore, the consequences of firearm misuse are more dire in Kenya than Uganda, hence few Kenyan officers could attempt to break the firearm code compared to security officers in Uganda [106].

The present study also found a statistically significant difference between the country of origin and the forms of suicide/homicides. Uganda had a significantly higher suicide rate among security forces than Kenya. However, Kenya had a higher national suicide rates of 6.5 per 100,000 in 2021 (an increase from 6.1 per 100,000 in 2019) [107, 108], than Uganda (with a national suicide rate of 4.6 per 100,000 in 2019) [108]. Our study identified only suicides by shooting among security forces, which might have caused this discrepancy since majority of other forms of suicide were not reported. In addition, a recent study in Kenya found that over 50% of suicides recorded in hospital health records occur by poisoning which contributed significantly to the national suicide rate, compared to the contribution by suicide by shooting [109]. In Uganda, suicide by use of poison has also been noted to be the commonly used method [110, 111] among security forces and general population. Although, studies in Uganda have reported suicide by shooting/use of firearm among the commonly used methods of suicide [112]. However, these are just observational trends, and establishment of such suicide database would be important to determine the true trends.

### Gender of the perpetuators

Perpetuators were mainly male officers with about 5% being females, corresponding to a female: male ratio of 1: 18 in the present study. Historically, this is the widespread report worldwide where the ratio of males who die by suicide or involved homicides in the general population is always greater than females. Among the suicides in this study, the ratio of females to males was 1: 13. In a similar study analyzing the content of media reports about suicides among police officers in Ghana, the suicide ratio of females to males was 1:14.6, which is in agreement with findings of this press media review study [113].

Some studies suggest that among male police officers, high community expectations and the notion that police officers have to be brave/tough to deal with all painful situations, might have contributed to chronic stress and poor copying strategies among security officers resulting into mental health problems which precipitate into suicide [113]. The cultural aspects of manhood in African perceives men as strong, masculine and providers, where in all aspects of their responsibility, they are not supposed to fail [17]. In case of failure or severe distress, males are more likely to complete suicide through the use of more lethal methods (like firearms – guns) since failure makes them feel less of a man [17].

### The age of perpetuators and forms of suicide/homicides

Perpetuators who died by suicide were significantly older, and this is consistent with similar studies that reported suicide and suicidal behavior to increase with increase in age [114, 115]. Older security officers may have cumulative stressors over their life time like financial stress resulting from a high number of dependents, increased responsibilities and cumulated job-related trauma which may precipitate into suicide [114, 116]

Perpetrators involved in homicides were younger than those who died by suicide. We believe that this may be related to less job experience in handling stressors among the public and less control in handling firearms [117].

### Number of victims lost during the incidents

Most incidents led to the loss of one victim. This is because these incidents resulted from personal or one-on-one misunderstandings with no intention to hurt anyone else. These were spontaneous and unplanned acts following heated arguments that may have led to loss of impulsive control, no wonder the potential causes from brief incident descriptions were related to mostly personal reasons such as financial disagreements and relationship dysfunctions.

Security officers who were involved in homicides involving more than one victim were statistically significantly older than those who killed one. Such perpetrators may have complex motives and pre-planned opportunities to end their victims. However, this relationship requires further investigation with bigger databases.

### Security officers (including perpetrators and victims)

About 4 out of 5 perpetrators were non-commissioned government employees. The non-commissioned officers may be less experienced with use and handling of fire arms, younger, potentially work for longer hours in harsher conditions and earn lesser pay compared to commissioned security officers [118]. These various factors may interplay/influence homicides/homicide-suicide activities among non-commissioned security officers. All security officers should uphold their constitutional mandates and the welfare for non-commissioned officers should be improved to prevent occurrence of such incidents.

### Mental illness among perpetuators

In the present study, only 2 perpetuators had history of mental health-related illness. From the brief incident descriptions, poor financial conditions and relationship dysfunctions among others were the predominantly potential stressors other than mental illness.

Their mental illness may be related to their professional work demands that involves long deployments with involuntary disconnection from the family and friends. Potential occupation hazards like disrupted sleep cycles due to wakeful watching to protect against security threats and handling of offenders who are potentially harmful to the security officers [119]. Involvement in keeping law and order among mobs of dangerous offenders may involve physical and psychological violence where some police officers may be permanently injured or lose lives [119].

In most cases, the standards of living are poor with less remuneration to afford basic life standards. The poor living conditions, community demands, occupational hazards, high number of dependents, and the feeling of failing their own families, may worsen or lead to depressive-like symptoms and anxiety-like symptoms. For better mental health and prevention of mental illness related homicides/suicides/suicide-homicides, emphasis should be put on improving their standards of living and addressing their occupational hazards [4].

Training in the armed forces is aimed to create resilient personnels with an appropriate subjective security judgement. They are trained to have a mental fortitude to face averse situations head-on in line of duty [120]. Apart from equipping them with knowledge, functions, processes of the country’s legal system, they are trained in the use of firearms, which becomes part of their self-defense mechanisms. Therefore, they tend to assume a ‘higher level of power’ in the community [120]. After training, most security officers receive duty guns for security maintenance at duty. With time, ‘pulling-a-trigger’ becomes automatic in times of threat. Therefore, they are more inclined to opening fire which may result in severe injuries and death of victims.

Additionally, studies among security forces report substance abuse, especially alcohol use and dependency, use of stimulants (like caffeine and nicotine). Studies have shown that police officers may use high dose caffeine and tobacco smoking due to fatigue, stress, and burnout in order to prevent or promote sleep [121]. This may result into substance use disorders and other associated disorders that may disrupt one’s judgement, resulting into shooting. Although, we used grey data from press media reports in which most of this information is not fully clearly captured, there was a remarkable number of perpetuators who were alleged to have been drunk while others were in bars and entertainment places, which are hotspots of substance abuse in the communities.

Evidence also suggests that army/police officers are likely to exhibit the “dark triad” of personality traits i.e., narcissism, psychopathy and machiavellianism which have been correlated with antisocial behavior, distrust of others, exploitation, manipulativeness, social malevolence, emotional coldness, duplicity, aggressiveness and substance abuse [122–124]. This may contribute to loss of impulsive control resulting into shooting.

### Criminalization of mental illness in homicides, suicides, and homicide-suicides

Due to the fact that all perpetrators didn’t have noticeable occupational dysfunctions, it is less likely that these incidents were due to mental disorders. It should be noted that suicide is not only caused by severe mental illness. However, as discussed above, welfare must be considered holistically if we want to understand the possible triggers of mental health related illness especially among at-risk populations like uniformed personnels.

### Study implications

This study creates insightful discussions about the fate of national security for both countries. This is based on the fact that promoters of law and order have potential to cause insecurity, which calls for quick, effective and sustainable solution. In addition, security officers being perpetuators impacts on the public perception and trust in security agencies/bodies. This in itself might promote insecurity due to the portrayed vulnerability among security personnels. We also need to appreciate that security officers are fellow members of the community with a superior conviction for patriotism, who need better welfare. Mental health awareness, prevention, and screening of mental health problems among security officers would prevent such emotional reactions that perpetuate into violence. Besides creating awareness, this study is a foundation to many future studies which should answer the “why” regarding the rising number of suicides, homicides, and homicide-suicides among security officers.

### Study limitations

There are a few limitations that should be considered when interpreting the results of the current study. Firstly, not all suicides, homicides, homicide-suicides are reported by press media, and thus this is likely to underestimate their true incidence among security officers. In addition, there may be bias in the incidences reported (e.g., tendency to report incidences of high-ranking security officers which are ‘newsworthy’). Therefore, there are a minority of incidents that may not be reported by the press media resulting into further underestimation. Secondly, data collected is totally reliant on information provided in the press media reports some of which may not be totally accurate (e.g., the brief incident descriptions). However, we merged more than one incident report available to triangulate descriptions of the same incidence from more than one press media website in order to maintain consistency in reporting. Thirdly, some incidents may have been omitted due to the press media following guidelines for reporting such sensitive incidences among security officers. Lastly, the number of incidents identified in this study were very few for generalizations.

### Conclusion

This study provides evidence from a community perspective (press media reports) about suicides, homicides, and homicide-suicides among security personnel, who are mandated to prevent them. Uganda stands on the worst side compared to Kenya, with Ugandan army involved more than the Ugandan police, and vice versa for Kenya. Potential triggers for these incidents are multi-factorial and inter-related, requiring holistic approach and individualization of preventive programmes to potential causes. In addition, routine screening of mental health problems to enable early interventions is recommended among security officers. But further research ought to be urgently conducted to qualitatively understand the actual causes of such incidents. In addition, epidemiological research needs to be conducted to better understand the possible distribution of psychological challenges among security officers.

### Supplementary Information

**Additional file 1: Supplementary table 1.** Characteristics of men-in-uniform involved in homicide-suicide, homicide and complete suicide.

**Additional file 2: Supplementary table 2.** Characteristics of victims who succumbed to suicide-homicides and homicides.

**Additional file 3: Supplementary table 3:** Service categories and the corresponding ranks.

**Additional file 4.** Dataset.

## Data Availability

The dataset used/or analyzed during the present study have been availed as a supplementary file (Dataset).
