# Supplementary material for: Firearm-related suicides, homicides, and homicide-suicides involving security officers in two East African Countries: a press media review
Source: BMC Psychiatry. 2023 Nov 24;23:877. doi: 10.1186/s12888-023-05368-6 (PMC10675850; doi:10.1186/s12888-023-05368-6)
Supplement: Supplementary file 1 — Additional file 1: Supplementary table 1. Characteristics of men-in-uniform involved in homicide-suicide, homicide and complete suicide. [file 12888_2023_5368_MOESM1_ESM.docx]

**Supplementary table 1: Characteristics of men-in-uniform involved in homicide-suicide, homicide and complete suicide.**

| **Case code** | **Month, Year, Time of the day** | **Sex of security officer** | **Type of security (service categories) – rank; *Employer*** | **Form of offences** | **Lives lost** | **Brief incident description** |
| --- | --- | --- | --- | --- | --- | --- |
| 1 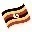 [39] | May, 2023, Day | M | PO (J) – Constable; ***G*** | Homicide | 1 | Perpetrator with history of mental illness had moved with the gun to the loan agency illegally. When they had an unresolving disagreement over loan repayment terms with victim, perpetrator decided to open fire and killed the victim on spot. |
| 2 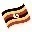 [15-17] | May, 2023, Day | M | PO (J) – Constable; ***G*** | Homicide | 1 | While at security officer’s workplace, the victim abused the security officer while taking photos with a woman, triggering a heated argument resulting into shooting of the victim by security officer. |
| 3 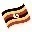 [40] | May, 2023, Night | M (47 years) | PO (J) – Constable; ***G*** | Suicide | 1 | The security officer (perpetrator) first aimed and shot at his superior’s house (no one was harmed), and later turned the gun on himself. |
| 4 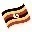 [41, 42] | May, 2023, Day | M (26 years) | SG; ***P*** | Homicide | 1 | An argument erupted between the two guards (perpetuator and victim), and shortly after a gunshot was heard coming from their room they shared, when the victim had been shot dead. |
| 5 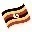 [43, 44] | May, 2023, Day | Married, M | AO (J); ***G*** | Suicide - homicide | 1 | While at home in the morning, the bodyguard to the victim, shot dead the victim at close range as he was entering his vehicle to go to work citing allegations of delayed salary payments. Immediately, bodyguard flee from scene and entered a nearby salon where he shot himself dead. |
| 6 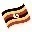 [45] | May, 2023, Day | M | SG; ***G*** | Suicide | 1 | After working at the new night duty station for 2 days, he was found dead in pool of blood in morning at duty. A friend had earlier heard him cocking the gun that morning. |
| 7 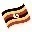 [46, 47] | May, 2023, Day | M | SG; ***P*** | Homicide | 1 | Two security guards (offender and victim) were involved in a heated argument that was later followed by a shooting. Both were working well together on duty that morning before the shooting. |
| 8 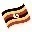 [48, 49] | April, 2022, Night | Single, M (36 years) | PO (S) – Assistant Superintendent of Police; ***G*** | Homicide | 1 | The victim had parked his car waiting for a friend. The car was hit by another car operated by the perpetuator. This resulted into a disagreement which prompted the perpetuator to shoot and kill the victim on the spot. |
| 9 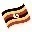 [50] | April, 2022, Day | M | PO (J) – Constable; ***G*** | Suicide | 1 | After having breakfast with his friends, the perpetrator went to his residential. He looked stressed recently. A friend who was asked to deliver an item to the victim found his dead body in a pool of blood with a gun pointing around his neck. |
| 10 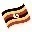 [51] | April, 2022, Night | Married, M (27 years) | AO (J) – Private; ***G*** | Suicide – homicide | 2 | The perpetuator reportedly came with a motive, entered their house, and proceeded to their bedroom, where he found his fiancé. He shot her in the chest causing her instant death, and he shot himself through the eye. |
| 11 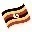 [52] | April, 2022, Day | M (45 years) | PO (N); ***G*** | Suicide | 1 | Police officer returned home from work at 10:00am for breakfast and had locked himself in his residential. At 1:00pm gunshots were heard from his room, and when the door was hit-open, they found him in a pool of blood, with gun on his chest. |
| 12 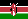 [12-14] | March, 2023, Night | Married, M | PO (S) – Corporal; ***G*** | Suicide - homicide | 3 | The perpetuator excused himself from his night duty and came back to his home to allegedly to pick a jacket. The neighbour (2^nd^ victim –wife to perpetuator’s friend) heard commotion from their house and had come to check on them where he was shot dead by the perpetuator before locking himself in with his girlfriend. He shot dead his girlfriend and later shot himself through the neck. |
| 13 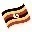 [53, 54] | March, 2023, Day | Married, M | AO (S) – Lance corporal; ***G*** | Homicide | 2 | Perpetuator found in a bar a group of men (including the male victim) and a woman with whom was in relationship (the second victim), and interrupted them leading to a heated argument. He left with a promise to return with vengeance. At a distance, he opened fire towards them, after instructing one to leave the crowd, he shot and killed one male on spot and severely injured the woman causing her death later. |
| 14 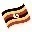 [55] | February, 2023, Day | Married, M | AO (S) – Captain; ***G*** | Suicide | 1 | After abruptly halting his work trip, he returned home, pensive and restless, he requested for a pen and paper, and entered the house. A gun shot was heard from the house, and his corpse laid motionless with an alleged suicide note citing a long-standing torment from workmates that had caused his high blood pressures too. |
| 15 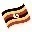 [56] | February, 2023, Night | M | SG; ***P*** | Homicide | 1 | The perpetuator picked a fight with the UPDF officers at a bar. Initially, the victim disarmed the perpetuator giving a gun to another security guard. Perpetuator attacked this security guard and restored back the ammunition which he used to kill the victim. |
| 16 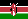 [57] | December, 2022, Day/Night | M | PO (J) – Constable; ***G*** | Suicide - homicide | 2 | After carrying him to the airport, the boda-boda operator (victim) and police officer (perpetrator) had a heated argument about the payments that that caused the perpetuator to open fire. Later, perpetuator was also found dead at a thicket after fatally shooting himself under the chin. |
| 17 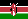 [58] | November, 2022, Day | F | PO; ***G*** | Suicide | 1 | After sharing a suspicious suicide post on her phone status, a day earlier, a fellow officer heard a loud noise from her house where she was found dead with a fire arm on the sides. |
| 18 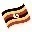 [59] | November, 2022, Day | M | AO; ***G*** | Homicide | 1 | While exiting from a school, the perpetrator found overcrowding of vehicles and boda-bodas at the school gate, and he squeezed his car on the sides where victim had parked resulting into an altercation causing public concern. Perpetrator picked his gun and shot dead the victim. |
| 19 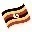 [60] | October, 2022, Day | M (21 years) | PO (J) – Constable; ***G*** | Homicide | 1 | With a prior motive, the perpetuator entered into the shop belonging to the victim. He aimed the gun to the victim, shot him dead in the chest, and the perpetrator tried to run away but was intercepted by the people and arrested. |
| 20 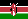 [61] | October, 2022, Night | Married, M | AO; ***G*** | Suicide - homicide | 2 | Perpetrator was on duty and asked to go for food, he decided to visit his girlfriend instead. After locking themselves inside the house, a local resident heard the sound of a gunshot from their house. Their lifeless bodies found in a pool of blood with perpetrator still dressed in military combat with entry gunshot wound on chin exiting at back of the head. |
| 21 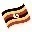 [62] | October, 2022, Day | Married, M | PO; ***G*** | Suicide | 1 | The previous night, the perpetrator knocked on houses of his friends as if he had something chasing him, after which he was escorted back to his home. At 9am, his wife heard the sound of a gunshot from their house, only to find him dead in a fresh pool of blood on their bed with the pistol in his hand. |
| 22 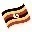 [63, 64] | September, 2022, Day | M (27 years) | PO (J) – Constable; ***G*** | Suicide - homicide | 2 | The perpetrator and victim were friends and chronic alcohol users who had been drinking from morning to mid-day at a neighbour’ s home before a disagreement sparked among the two. Their neighbour sensed danger and chased them from his home. Moments later, while still arguing the perpetrator shot and killed the victim. |
| 23 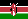 [65] | September, 2022, Day | Married, M | PO (S) - Deputy commanding officer; ***G*** | Suicide - homicide | 2 | After 3-months of intimate relationship dogged with brawls, the perpetrator shot and killed the alleged lover as he had escorted her about 100 meters to her house, accusing her of running other affairs. The perpetrator later turned the gun on self, dying instantly. Both bodies were found in a pool of blood along the roadside. |
| 24 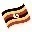 [66] | August, 2022, Day | Married, M (45 years) | PO (S) – AIP; ***G*** | Suicide | 1 | Just after hours of arriving at a new station, the perpetrator locked himself inside the new home. His wife reported to the barracks how her husband isn’t picking calls, only to find him in a pool of his blood on a mat. |
| 25 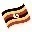 [67] | August, 2022, Night | M | AO (J) – Private; ***G*** | Suicide | 1 | While in his tent, the victim’s friend heard a gunshot and fled away thinking that they were attacked. The lifeless body of victim was found in his tent in a pool of blood with a gun on his chest and a radio call on. |
| 26 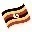 [68] | July, 2022, Day | M (27 years) | SG; ***G*** | Homicide | 1 | The perpetrator allegedly accused his workmate (victim) for coming late at work in the afternoon. This precipitated into shooting and killing the victim instantly. |
| 27 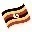 [69] | June, 2022, Day | Married, M (27 years) | Others (N) – Prisons warden; ***G*** | Suicide - homicide | 2 | The perpetrator (prisons warder) had been in an intimate relationship with his girlfriend (prisons wardress) who had come to pay him a visit. However, while on duty with her visiting girlfriend, the perpetrator shot and killed her, and he then shot himself dead on the head. |
| 28 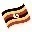 [70] | June, 2022, Day | M | AO (J) – Private; ***P*** | Homicide | 1 | The two UPDF soldiers (perpetrator and victim) had a long-standing conflict over the house maid at the home where they were deployed to guard. This sparked off the fight that led to shooting. The perpetrator fled the scene. |
| 29 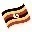 [71] | May, 2022, Day | Married, M | AO (S) – Lance corporal; ***G*** | Homicide | 1 | While UPDF major (victim) briefing his juniors (nearing his retirement) on the parade, the perpetrator came late for work, and requested to join the parade. Later, perpetrator UPDF officer asked for permission to take his family to village, even during public transport restrictions of COVID-19. Perpetuator had for long lamented on how victim intentionally always deployed him away from his family. Permission wasn’t given immediately, but promised on following day. This later precipitated into shooting. |
| 30 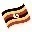 [72] | March, 2022, Night | Married, M | PO; ***G*** | Homicide | 3 | The police officer (perpetrator) suspected that victim was involved in a love affair with his lover, a bar maid, after finding him buying local beer for her. When the victim left, the perpetrator followed him to a fuel/gas station where he shot and killed him, the station security guard, and another turn-man/casual laborer of a nearby motor vehicle. |
| 31 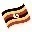 [73] | February, 2022, Day | Married, M | AO (S) – Captain; ***G*** | Suicide | 1 | The high-ranking UPDF officer (victim) returned home at 3pm and entered the house saying he wanted to take a nap. A few minutes later, the wife heard the sound of a gunshot inside the house, only to find the victim dead with a suicide note near the body, notifying people about his death and detailing the reasons why. |
| 32 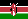 [74, 75] | January, 2022, Day | Married, M (27 years) | PO (J) – Constable; ***G*** | Suicide - homicide | 2 | The victim had travelled from his station to pick up her phone that he (perpetrator) had allegedly snatched from her on suspicion that she was having an affair. He locked her inside the house, and later gunshots were heard. After breaking-in, the two lifeless bodies were found in a pool of blood in the perpetrator’s house. |
| 33 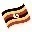 [76, 77] | January, 2022, Night | M | PO (S) – Corporal; ***G*** | Suicide | 1 | It is alleged that the police officer shot himself dead at about 4am inside his place of resident, under unclear circumstances. |
| 34 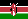 [78] | 2022, Night | M (29 years) | PO; ***G*** | Suicide - homicide | 2 | The police officer (perpetuator) was on night duty but requested permission from work saying he was feeling unwell, and was released home. He didn’t sign off or hand over his gun. His wife (victim) was expectant. The two got into an argument in the middle of night, prompting the perpetuator to shoot 16 bullets at her pregnant wife, before shooting himself dead. |
| 35 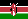 [79-81] | December, 2021, Night | Married, M | PO (J) – Constable; ***G*** | Suicide - homicide | 6 | Under unclear circumstances, the perpetrator went on a rampage after 12am, where he shot dead his wife and directed the fire arm to a boda-boda rider, and two other civilians who died instantly, before shooting himself dead in his house. |
| 36 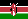[82] | December, 2021, Day | M | PO (S) – Corporal; ***G*** | Homicide | 1 | The police officer (perpetrator) was reprimanded by the officer-in-charge (victim) for coming late for work and seemingly drunk. He took offence, got angry, shot the victim 5 times killing him instantly, and he fled the scene, but was later rounded up at an entertainment joint. |
| 37 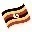 [83] | November, 2021, Night | M | PO (J) – Constable; ***G*** | Suicide | 1 | The police officer at aviation police is believed to have shot himself dead with a bullet wound that went through the chin to the head. He left a suicide note addressed to his mother. |
| 38 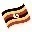 [84] | November, 2021, Night | Married, M | PO (S) – Corporal; ***G*** | Homicide | 1 | The perpetrator has been in a relationship with the victim for 5 years. Following unknown circumstances, he stormed the home of his fiancé at 10pm and shot her dead, after which he abandoned the vehicle and the gun he used to kill and attempted to flee, but was arrested before he fled. |
| 39 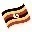 [85] | September, 2021, Night | M (45 years) | AO (N) – Police trainer (Pt); ***G*** | Suicide - homicide | 2 | The perpetrator followed the victim (colleague’s wife) into the shelter where she had gone to take a bath at about 9pm and shot her dead, then turned the gun to himself and died instantly. This happened when the husband to the victim and other officers were drunk at a nearby trading centre. |
| 40 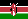 [86] | August, 2021, Day | Married, M | PO (S) – Corporal; ***G*** | Suicide - homicide | 2 | The police officer (perpetrator) had a long-standing dysfunctional relationship and over-protective of his girlfriend (victim). He fractured his girlfriend’s leg in a fight, who was taken to hospital. He told some people that he will be going somewhere far and will never return. He called her sick girlfriend and told her “I am about to kill myself”. But before, the perpetrator abruptly showed up at the hospital, and shot her sick girlfriend dead. He then visited his working police station and shot aimlessly. On attempting to disarm him, he turned on the gun and shot himself through the chin and bullet exited on upper forehead, and died instantly. |
| 41 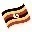 [87] | August, 2021, Night | Married, M | SG; ***P*** | Homicide | 1 | Under unknown circumstances, the perpetrator shot dead his girlfriend (Congolese national) killing him instantly. |
| 42 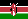 [88-90] | July, 2021, Day/Night | F (34 years) | PO (S) – Corporal; ***G*** | Suicide-homicide | 3 | The perpetrator was re-known for her involvement in multiple love affairs mostly with police officers and with tragic endings. In the current incident, the perpetrator killed 2 people in 24 hours. She first shot and killed her fellow police officer in his car at police station, before luring the second victim into a hotel where she shot him in head and flee from hotel at mid-night. Later, the perpetrator shot herself in the head in her parents’ home in bathroom, where her lifeless body was discovered. |
| 43 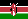 [91, 92] | April, 2021, Night | Married, M | PO (S) - General Service Unit (GSU) officer; ***G*** | Suicide - homicide | 2 | The perpetrator, a GSU officer, and husband to victim (female traffic officer) had an argument about moving out of GSU camp where she had moved recently. The argument started when he found her taking supper in the new residence, which prompted him to move out before walking back and shot the woman in the chest eight times. He turned the gun on his chin bursting his head. |
| 44 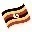 [93] | April, 2021, Night | M | AO (S) – Major; ***G*** | Homicide | 1 | The perpetrator is alleged UPDF spy who reportedly shot dead a civilian male over a disagreement about a woman in an illegally operated bar. This happened shortly into the lockdown when operation of bars and other public places had been closed. The perpetrator fled from the scene. |
| 45 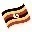 [94] | April, 2021, Night | M | Others (N) – LDU officer; ***G*** | Homicide | 1 | As the victim was returning from the village after the brothers burrial, he hurriedly entered the late’s home which was being guarded by LDU personnel after he suspected that some people were following him and he knocked the gate with his motor vehicle while gaining entrance. This triggered a disagreement that led to shooting of victim by the LDU officer. |
| 46 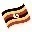 [95] | February, 2021, Day | Married, M | AO (J) – Private; ***G*** | Suicide | 1 | Under unclear circumstances, gunshots were heard from the perpetrator’s tent at around 6pm only to find him lifeless with a shuttered head. His colleagues might be a result of a family misunderstanding. |
| 47 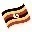 [96] | January, 2021, Day | M | AO (S) – Corporal; ***G*** | Homicide | 4 | Under unclear circumstances, but alleged to be under influence of alcohol, the perpetrator shot and killed two LDU officers, one civilian and one police special constable. The civilian was killed when the perpetrator entered civilian residences during the gunfight. The perpetrator was also gunned down in self defence and to stop his wanton action. |
| 48 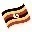 [97] | August, 2020, Night | M | PO (J) – Private; ***G*** | Homicide | 1 | The perpetrator was allegedly hired by victim’s brother. This followed a long-standing family land dispute that prompted the brother to hire the perpetrators who called the victim out of the victim. When he later opened the door to go out of the house, he was shot dead, and was lying in a pool of blood and fighting for his life, and later died on the way to hospital. |
| 49 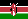 [98] | December, 2020, Night | Married, M | PO (J) – Constable; ***G*** | Suicide - homicide | 2 | Under unclear circumstances, the police officer (perpetrator) and his wife were found dead in their house with gunshot wounds on their bodies which were lying in a pool of blood. |
| 50 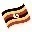 [99] | December, 2020, Night | M | AO; ***G*** | Homicide | 3 | The perpetrator is said to have knock at the home of 2 of 3 victims saying that he had a pregnant woman. When they refused to open for him, he went to the window where victims were sleeping and he started shooting killing the them. The 3^rd^ victim was killed after he shot in the air, when the victim rushed to switch off the lights in her house but the gunman shot at her through the window. |
| 51 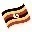 [100] | February, 2020, Day | M | PO (J) – Constable; ***G*** | Homicide | 2 | The perpetrator and fellow police officer (1^st^ victim) had a heated argument on how to proceed with a case. The perpetrator picked his gun and shot at victim several times killing him instantly. The 2^nd^ victim was an LDU personnel who sustained serious injuries and was pronounced dead upon reaching the hospital. |
| 52 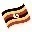 [101, 102] | February, 2020, Day | M | AO (S) – Corporal; ***G*** | Homicide | 3 | The perpetrator had misunderstandings with the victims’ family whom he accused of grazing their goats on his land. In additionally, it is alleged that the division chairman attempted to resolve the dispute but the outcomes of the mediation angered the perpetrator, which led to the shooting. |
| 53 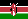 [103] | January, 2020, Day/Night | M | PO; ***G*** | Suicide - homicide | 2 | While at a bar in the night, the victim engaged in a fistfight with one police officer (perpetrator) over unknown cause. Other police officers arrested the victim and frog-marched him to the nearby police post. The perpetrator pursued them, took a gun and shot victim dead while he was reporting at the desk. He was found dead in morning and that it appeared he had shot himself. |
| 54 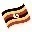 [104] | November, 2020, Day | M (40 years) | AO – Corporal; ***G*** | Suicide | 1 | The perpetrator went missing after a morning exercise with 120 rounds of ammunition and a search for him was launched. Later, information was got that he had shot himself under unknown circumstances. |
| 55 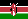 [105] | November, 2020, Night | Married, M | Others - Kenya Forest Service officer; ***G*** | Suicide – homicide | 2 | After having an alleged restrained relationship over time, the perpetrator came back home and picked a quarrel with his wife (victim) at about 8pm for unknown reasons. It was then that the perpetrator took his gun and shot his wife in the head, killing her on the spot, and then turned the same gun on himself. Both bodies were found lifeless in pools of blood outside their rented house. |
| 56 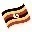 [106] | November, 2020, Day | M | AO; ***G*** | Homicide | 1 | The UPDF officer (victim) was pursued by perpetrators at a local church where he was first shot at while in legislators’ car. On attempt to exit the car, his assailants reportedly pursued him and fatally shot him dead in the church compound causing panic among congregants. |
| 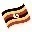 = Uganda; 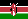 = Kenya; M = Males; F = Females; AO = Army officers, PO = Police officer, SG = Security guard; N = Non-commissioned; J = Junior; S = Senior; ***P =*** Private, ***G*** ***=*** Governemnt; LDU = Local Defense Unit; GSU = General Service Unit; UPDF = Uganda People’s Defense Force; COVID-19 = Coronavirus disease – 2019;  “Cases 2, 4, 34, 56, 2 were privately employed whereas all other cases were government civil workers” | | | | | | |
