# Supplementary material for: Firearm-related suicides, homicides, and homicide-suicides involving security officers in two East African Countries: a press media review
Source: BMC Psychiatry. 2023 Nov 24;23:877. doi: 10.1186/s12888-023-05368-6 (PMC10675850; doi:10.1186/s12888-023-05368-6)
Supplement: Supplementary file 2 — Additional file 2: Supplementary table 2. Characteristics of victims who succumbed to suicide-homicides and homicides. [file 12888_2023_5368_MOESM2_ESM.docx]

**Supplementary table 2: Characteristics of victims who succumbed to suicide-homicides and homicides**

| **Victim’s corresponding cases** | **Sex (Age)** | **Occupation of victim** | **Official category** | **Relationship of victim with security officer** | **Place of death** |
| --- | --- | --- | --- | --- | --- |
| 1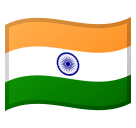 | M | Money lender | Civilian | - | At work |
| 2 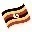 | M | UPDF officer (Lance Corporal) | Officer | - | At work |
| 4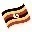 | M | Security guard | Officer | Workmate | At work |
| 5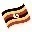 | M (68 years) | UPDF officer & Former cabinet minister | Officer | Minister’s bodyguard | At home |
| 7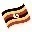 | M | Security guard | Officer | Workmate | At work |
| 8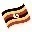 | M | - | - | - | In community |
| 10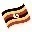 | F | - | - | Fiancé | At home |
| 12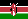 | F | - | - | Girlfriend | At home |
| 12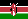 | F | - | - | Wife to perpetrator’s friend | At their home |
| 13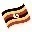 | M | - | - | - | Entertainment place |
| 15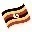 | M | UPDF officer | Officer | Workmate | Entertainment place |
| 16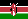 | M | Boda-boda rider | Civilian | - | In community |
| 18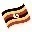 | M (38 years) | Boda-boda rider | Civilian | - | In community |
| 19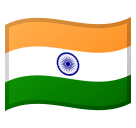 | M | Business man | Civilian | - | At work |
| 20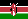 | F | - | - | Girlfriend | At home |
| 22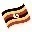 | M (47 years) | Police officer (corporal) | Officer | Workmate | Entertainment place |
| 23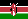 | F (30 years) | Bar maid | Civilian | Girlfriend | At home |
| 26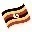 | F (28 years) | Security guard | Officer | Workmate | At work |
| 27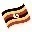 | F (24 years) | Prison wardress | Officer | Wife | At work |
| 28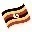 | M (29 years) | UPDF officer | Officer | Workmate | At work |
| 29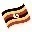 | M | UPDF officer (Major) | Officer | Officer-in-charge | At work |
| 30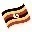 | M (25 years) | Farmer | Civilian | - | In community |
| 30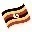 | M (45 years) | Security guard | Officer | - | At work |
| 30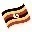 | M (19 years) | Casual laborer | Civilian | - | In community |
| 32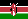 | M (25 years) | Police officer | Officer | Girlfriend | At home |
| 34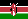 | F (27 years) | - | - | Wife | At house |
| 35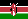 | F | - | - | - | At home |
| 35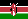 | M | Boda-boda rider | Civilian | - | In community |
| 35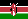 | M | - | Civilian | - | - |
| 35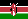 | M | - | Civilian | - | In community |
| 35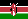 | M | Boda-boda rider | Civilian | - | In community |
| 36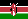 | M | Police officer (Sergeant) | Officer | Officer-in-charge | At work |
| 38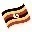 | F | - | - | Fiancé | At home |
| 39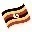 | F | - | - | Colleague’s wife | At home |
| 40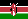 | F (29 years) | - | - | Girlfriend | Other***** |
| 41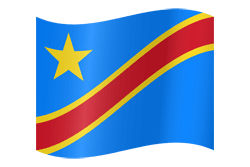 | F (33 years) | - | - | Girlfriend | - |
| 42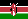 | M | Police officer (constable) | Officer | Lover | At work |
| 42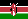 | M (32 years) | Businessman | Civilian | Lover | Entertainment place |
| 43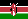 | F | Traffic officer | Officer | Wife | At home |
| 44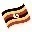 | M (43 years) | - | - | - | Entertainment place |
| 45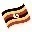 | M | Journalist | Civilian | - | In community |
| 47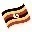 | M | Police officer (constable) | Officer | Workmate | - |
| 47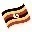 | M | LDU personnel | Officer | Subordinate | - |
| 47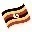 | M | LDU personnel | Officer | Subordinate | - |
| 47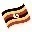 | F | - | Civilian | - | At home |
| 48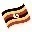 | M | Farmer | Civilian | - | At home |
| 49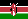 | F (28 years) | - | - | Wife | - |
| 50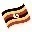 | M (22 years) | - | - | - | At home |
| 50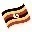 | F (26 years) | - | - | - | At home |
| 50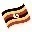 | F (50 years) | - | Civilian | - | At home |
| 51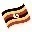 | M | Police officer (corporal) | Officer | Workmate | At work |
| 51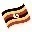 | M | LDU personnel | Officer | - | - |
| 52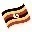 | F (52 years) | - | - | Neighbour | At home |
| 52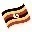 | M (20 years) | High school student | Civilian | Neighbor’s son | At home |
| 52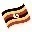 | M (18 years) | - | Civilian | Neighbor’s son | At home |
| 53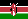 | M | - | Civilian | - | At police post |
| 55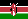 | F | - | - | Wife | At home |
| 56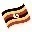 | M (24 years) | UPDF officer | Officer | Body guard | In community |
| 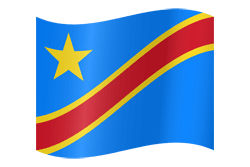 = Congolese; 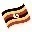 = Ugandan; 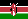 = Kenyan, 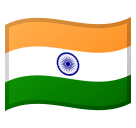 = Indian; M = Males; F = Females; LDU= Local defense unit; UPDF = Uganda Police Defense Force; Other* = Was shot and killed on hospital bed. | | | | | |
