# Supplementary material for: Firearm-related suicides, homicides, and homicide-suicides involving security officers in two East African Countries: a press media review
Source: BMC Psychiatry. 2023 Nov 24;23:877. doi: 10.1186/s12888-023-05368-6 (PMC10675850; doi:10.1186/s12888-023-05368-6)
Supplement: Supplementary file 3 — Additional file 3: Supplementary table 3: Service categories and the corresponding ranks. [file 12888_2023_5368_MOESM3_ESM.docx]

**Supplementary table 3: Service categories and the corresponding ranks**

| **Service categories** |  | **Cadre category** | **Police** |
| --- | --- | --- | --- |
| **Non-commissioned security officers** | Junior | -Private  -Lance corporal  -Surgent | - General service unit officer  -Prison warden  -Corporal  -Surgent  -Station surgent  -Head constable  -Constable |
|  | Senior | -Staff surgent  -Warrant officer 2  -Warrant officer |  |
| **Commissioned security officers** | Junior officers (Army)/Inspectorate officers (Police) | -Second lieutenant  -Lieutenant  -Captain | -Assistant Inspector of Police  -Inspector of Police |
|  | Senior officers (Army)/Gazetted officers (Police) | -Major  -Lieutenant colonel  -Colonel  -Brigadier  -Lieutenant general  -General | -Assistant Superintendent of Police |
| **Others** | Local defence unit (LDU), Security guards | | |
